# Observation and Management of Juvenile Myelomonocytic Leukemia and Noonan Syndrome-Associated Myeloproliferative Disorder: A Real-World Experience †

**DOI:** 10.3390/cancers16152749

**Published:** 2024-08-02

**Authors:** Bryony J. Lucas, Jeremy S. Connors, Heping Wang, Shannon Conneely, Branko Cuglievan, Miriam B. Garcia, Rachel E. Rau

**Affiliations:** 1 Department of Pediatrics, Baylor College of Medicine, Texas Children’s Cancer and Hematology Center, Houston, TX 77030, USA; 2 Department of Pediatrics, The University of Texas MD Anderson Cancer Center, Houston, TX 77030, USA; 3 Department of Bioinformatics and Computational Biology, The University of Texas MD Anderson Cancer Center, Houston, TX 77030, USA; 4 Department of Pediatric Oncology, Children’s Memorial Hermann Hospital, Houston, TX 77030, USA; 5 Department of Pediatrics, Ben Towne Center for Childhood Cancer Research, Seattle Children’s Hospital, University of Washington, Seattle, WA 98105, USA

**Keywords:** Juvenile Myelomonocytic Leukemia, monocytosis, myeloproliferative, Noonan syndrome, RASopathies, Noonan Syndrome-associated Myeloproliferative Disorder

## Abstract

**Simple Summary:**

Juvenile Myelomonocytic Leukemia (JMML) is a rare and clonal hematopoietic disorder of infancy and early childhood with myeloproliferative/myelodysplastic features resulting from germline or somatic mutations in the RAS pathway. Given its rarity, management is not standardized and varies widely, ranging from observation to bone marrow transplant depending on genomic and clinical features. We describe the course of JMML or Noonan Syndrome-associated Myeloproliferative Disorder in 22 pediatric patients treated at three institutions to provide guidance for monitoring versus intervention, including transplant, supported by patient outcomes. We provide additional insight into the expected time to spontaneous resolution in those with germline *PTPN11* mutations and treatment approaches for patients with germline *CBL* mutations where no standard exists.

**Abstract:**

Juvenile Myelomonocytic Leukemia (JMML) is a rare and clonal hematopoietic disorder of infancy and early childhood with myeloproliferative/myelodysplastic features resulting from germline or somatic mutations in the RAS pathway. Treatment is not uniform, with management varying from observation to stem cell transplant. The aim of our retrospective review is to describe the treatment and outcomes of a cohort of patients with JMML or Noonan Syndrome-associated Myeloproliferative Disorder (NS-MPD) to provide management guidance for this rare and heterogeneous disease. We report on 22 patients with JMML or NS-MPD managed at three institutions in the Texas Medical Center. Of patients with known genetic mutations and cytogenetics, 6 harbored germline mutations, 12 had somatic mutations, and 9 showed cytogenetic abnormalities. Overall, 14/22 patients are alive. Spontaneous clinical remission occurred in one patient with somatic *NRAS* mutation, as well as two with germline *PTPN11* mutations with NS-MPD, and two others with germline *PTPN11* mutations and NS-MPD remain under surveillance. Patients with NS-MPD were excluded from treatment analysis as none required chemotherapeutic intervention. All patients (5/5) treated with 5-azacitidine alone and one of the four treated with 6-mercaptopurine monotherapy had a reduction in mutant variant allele frequency. Transformation to acute myeloid leukemia was seen in two patients who both died. Among patients who received transplants, 7/13 are alive, and relapse post-transplant occurred in 3/13 with a median time to relapse of 3.55 months. This report provides insight into therapy responses and long-term outcomes across different genetic subsets of JMML and lends insight into the expected time to spontaneous resolution in patients with NS-MPD with germline *PTPN11* mutations.

## 1. Introduction

Juvenile Myelomonocytic Leukemia (JMML) is a rare and aggressive clonal hematopoietic disorder of childhood. Per the most recent WHO classification of tumors of the hematopoietic and lymphoid tissues (WHO-HEM5) and the International Consensus Classification (ICC) of Myeloid and Lymphoid Neoplasms, JMML is categorized as a RAS pathway activation-driven myeloproliferative neoplasm of early childhood [1,2]. Juvenile Chronic Myeloid Leukemia, Chronic Myelomonocytic Leukemia of Infancy, and Infantile Monosomy 7 Syndrome are all now classified under the name JMML [1,3]. JMML accounts for approximately 1–2% of all pediatric leukemias and has an estimated incidence of approximately 0.13/100,000 children 0 to 14 years of age per year [4,5,6,7]. In this study, we present the collective clinical and hematologic profiles, management, and outcomes of 22 pediatric patients with either JMML or Noonan Syndrome-associated Myeloproliferative Disease (NS-MPD), treated at the Texas Children’s Hospital, the MD Anderson Cancer Center, and the Children’s Memorial Hermann Hospital (hereafter collectively termed Texas Medical Center, or TMC).

### Background

JMML is characterized by the excessive production of myeloid progenitor cells and monocytes without maturation arrest during myeloid differentiation. These progenitor cells also show a high sensitivity to granulocyte–macrophage colony-stimulating factor in vitro, secondary to dysregulated activation of the RAS signaling pathway. As a result of the overproduction of myeloid cells and increased circulating monocytes, leukocytosis is common while other cell lines are suppressed, leading to anemia and thrombocytopenia [5]. The median reported age of presentation is 2 years, with 95% of cases being detected by 6 years of age and predominantly in males. Clinically, patients may present with malaise, fever, and/or skin findings, and almost all will have splenomegaly, often accompanied by hepatomegaly and/or lymphadenopathy on examination [6]. The clinical and laboratory diagnostic criteria of JMML as defined by the WHO and ICC are outlined in Table 1.

JMML is associated with mutations in the RAS signaling pathway, with nearly 90% of patients harboring either germline or somatic mutations of protein tyrosine phosphatase non-receptor type 11 (*PTPN11*), Casitas B-lineage lymphoma (*CBL*), Neurofibromatosis type-1 (*NF1*), neuroblastoma rat sarcoma (*NRAS*), or Kirsten rat sarcoma (*KRAS*) [9]. As such, JMML includes five recognized genetically and clinically distinct subtypes delineated by the driving mutation: JMML with somatically mutated *PTPN11*, germline mutated *CBL*, somatically mutated *NRAS*, and somatically mutated *KRAS* [10]. The cytogenetics in JMML patients are typically normal; however, monosomy 7 is the most common karyotype abnormality seen, present in approximately 25% of patients. Deletions of Chromosome 5q and 7q may also exist [5].

Patients with germline RASopathies, including Noonan Syndrome (NS), CBL syndrome, and Neurofibromatosis Type 1 (NF1), are at an increased risk for the development of JMML [11,12]. Germline *PTPN11* mutations are responsible for 50% of cases of NS. Children with germline *PTPN11* mutations are at increased risk for the development of a characteristically transient JMML-like myeloproliferative disorder in infancy classified as Noonan Syndrome-associated Myeloproliferative Disease (NS-MPD) [2]. While this entity often meets all the clinical criteria for JMML, it is thought to be polyclonal in origin [2] and it tends to have a mild course characterized by spontaneous remission. However, the time frame for resolution is variable and the indications for JMML-directed therapy are not clearly defined. A small subset of patients may benefit from low-intensity chemotherapy for the management of severe symptoms [9,13]. This mild course is in contrast to JMML driven by somatic mutations in *PTPN11*, which are reported in 35% of patients with non-syndromic, de novo JMML and require hematopoietic stem cell transplantation (HSCT) for the best chance of cure [14]. Patients with JMML secondary to germline *CBL* mutations may have spontaneous resolution of their disease [15]; however, more recent data suggest few actually do [16]; thus, the management of this rare subset of patients is still unclear. The incidence of JMML in patients with NF1 is increased more than 200-fold, and rarely, JMML can be the first presentation of NF1 [17]. Patients with germline *NF1* mutations generally require allogenic transplants for cure [18].

The clinical course of JMML is a varied spectrum. Patients with germline mutations associated with JMML may demonstrate a mild course, and approximately one-third of patients are able to achieve spontaneous, long-standing remission [6]. However, for patients requiring HSCT, the overall survival has been estimated at 64% [19]. Several features have been reported to be associated with the prognosis of JMML; however, the findings have been inconsistent across studies. Factors that may be associated with an unfavorable outcome include older age at presentation, elevated fetal hemoglobin, somatic mutations, complex cytogenetics, and relapse after HSCT [5,18]. A retrospective series published by the European Working Group of Myelodysplastic Syndromes in Childhood examined 110 children with JMML and established clinical predictors of poor survival or high-risk features at diagnosis: thrombocytopenia, age ≥ 2 years, and high fetal hemoglobin [20]. These findings were corroborated by a more recent large study of 119 patients with JMML who received the first HSCT between 2002 and 2021. Four distinct adverse prognostic factors impacting overall survival were identified: age > 2 years at diagnosis, ≥6 months from time to diagnosis to HSCT, monocyte count at diagnosis ≥7 × 10^9^/L, and the presence of additional genetic mutations [21].

At present, HSCT offers the only chance of cure for those patients who do not achieve spontaneous remission and is recommended for all children with *NF1* mutations and somatic *PTPN11* and *KRAS* mutations and most patients with somatic *NRAS* or *CBL* mutations [9]. In the pre-transplant period, therapy to reduce the disease burden is likely beneficial [16]. The optimal pre-transplant therapy regimen has not been clearly defined, but the most commonly used treatments incorporate an acute myeloid leukemia (AML) chemotherapy approach, which may include 5-azacitidine [22,23] and/or oral 6-mercaptopurine (6-MP). Conversely, close observation without treatment or low-intensity chemotherapy is recommended for patients with NS-MPD, germline *CBL* mutations, and specific somatic *NRAS* mutations [18].

Delineating between patients who may be observed versus those who require therapy, potentially including HSCT, is imperative to improve outcomes. While disease and clinical features associated with prognosis and response to therapy have been explored, the conclusions are limited by small patient numbers given the rarity of the disease. Thus, an ongoing collation of data is needed. In this study, we retrospectively review the collective TMC experience for children with JMML and NS-MPD. We describe their presentation, management, and outcomes along with examples of intervention.

## 2. Methods

### 2.1. Patient Selection

We conducted a retrospective chart review of all patients with a diagnosis of JMML from January 2010 to September 2023 who were treated at Texas Children’s Hospital and from January 2000 to September 2023 who were treated at MD Anderson Cancer Center and Children’s Memorial Hermann Hospital. Patients were identified through a search of the electronic medical record for the ICD-10 diagnosis code for JMML. We obtained discrete data including the patient’s diagnosis, age, sex, ethnicity, laboratory, and pathology results including diagnostic criteria for JMML, type and timing of treatment, and clinical outcomes including mortality. Patients with available sequencing data had targeting sequencing performed based on available testing at the time of treatment, either at the treating institution or commercial laboratories. This ranged from single-gene or limited-gene panels to current comprehensive genetic panels (Supplemental Table S1), testing known genes associated with hematologic malignancies. Response and remission status was defined as per Niemeyer et al. [24]. Institutional Review Board approval was obtained through each of the centers, and the requirement for informed consent was waived. Protected health information was initially collected; however, medical record numbers and names were replaced with study numbers after initial data collection was complete.

### 2.2. Statistical Analysis

Descriptive statistics were used to report patient characteristics, efficacy, and toxicity data. The duration of survival was defined from the time of diagnosis or the chemotherapy start date (whichever was earlier) to death or censored at the last follow-up. Fisher’s exact test was employed to assess the association between categorical covariates (sex, germline mutations, somatic mutations, abnormal cytogenetics, splenomegaly, chemotherapy, and HSCT) and survival status (alive/dead). The Mann–Whitney U test, a non-parametric test, was utilized to test significant differences between continuous covariates (age, blasts, platelets, and fetal hemoglobin) and survival status (alive or dead). The Pearson correlation test was employed to assess the relationship between two continuous covariates. Competing risk analysis was conducted using the Fine and Gray model to evaluate the associations of covariates with remission and death in the patients with JMML. The Cox proportional hazards regression model was employed to examine the relationship between covariates and time to death. Statistical analysis was conducted using Microsoft Excel (version 23.061.0319.0003), GraphPad Prism (version 10.0.3), and R (version 4.3.2).

## 3. Results

### 3.1. Patient Characteristics

#### 3.1.1. Patient Characteristics—NS-MPD

Four patients with NS-MPD were included in our study; their baseline characteristics are presented in Table 2. The median age at diagnosis was 1.4 months (range, 0–2 months). Half of the patients were female, and half were male. Patient caregivers self-reported patient race and ethnicity with two identifying as White non-Hispanic, one as Black, and one as Hispanic.

#### 3.1.2. Patient Characteristics—JMML

Eighteen patients with JMML were included in our study; their baseline characteristics are presented in Table 2. The median age at diagnosis was 15.7 months (range, 2–59 months). Eighty-three percent of patients were male (*n* = 15). Patients were of varying race and ethnicity as self-reported and documented in the medical record: 22% (*n* = 4) were White non-Hispanic, 56% (*n* = 10) were Hispanic, 11% (*n* = 2) were Black, and 11% (*n* = 2) were Asian.

### 3.2. Cohort Characteristics

#### 3.2.1. Cohort Characteristics—Patients Diagnosed with NS-MPD

**Figure 1:**
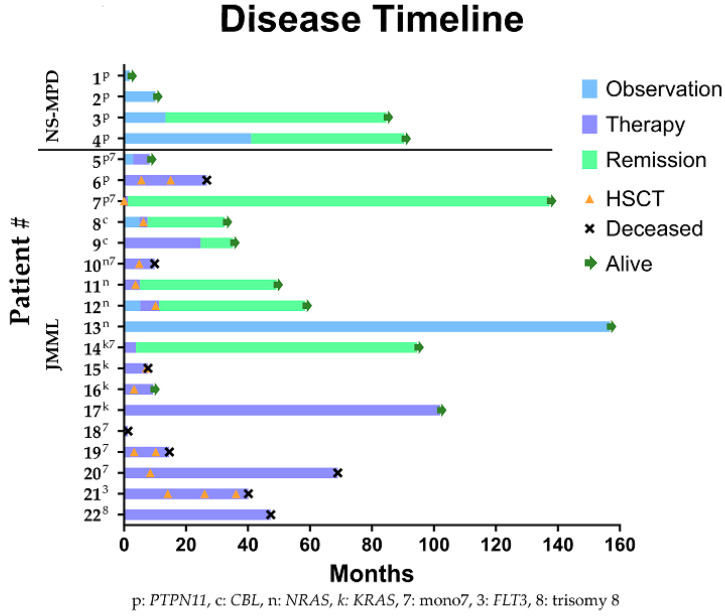
Swimmer plot showing individual patient clinical course over time. Each patient’s known major cytomolecular lesions are indicated by superscript text of each patient identifier number; p, *PTPN11*; c, *CBL*; n: *NRAS*, k: *KRAS*, 7, monosomy 7; 3: FLT3 mutation; 8: trisomy 8. Each bar represents the patient’s clinical course. Light blue represents time of observation without remission; purple bars represent time during which the patient was receiving therapy; green bars represent ongoing remission. Green arrows indicate ongoing survival. HSCT and death are indicated by orange triangles and black Xs, respectively. NS-MPD = Noonan Syndrome-associated Myeloproliferative Disorder, JMML = Juvenile Myelomonocytic Leukemia.

All four patients with NS-MPD had known germline mutations in *PTPN11,* and their cohort characteristics are included in Table 3 and Figure 1. Regarding the clinical diagnostic criteria, three of the four patients had documented splenomegaly. In the two patients who underwent bone marrow evaluations at diagnosis, both had less than 20% blasts, and all four patients had <20% peripheral blasts with one demonstrating no peripheral blasts. All patients had an absolute monocyte count (AMC) > 1 × 10^9^/L. Thrombocytopenia was noted in all four patients with NS-MPD. While fetal hemoglobin (Hb F) was elevated in all patients, levels were within the normal range for their newborn and infant ages.

No patients with NS-MPD received chemotherapy and none are deceased. Spontaneous remission occurred in two patients (times to clinical remission of 13.3 and 41 months) and two are in active surveillance without remission 2 and 10 months after diagnosis.

#### 3.2.2. Cohort Characteristics—Patients Diagnosed with JMML

The cohort and treatment characteristics of each of the 18 patients with JMML are presented in Table 3 and Figure 1. Of the patients with known molecular testing, two had germline mutations in *CBL.* Our cohort included a total of 17 known somatic mutations in 12 patients: 5 *KRAS*, 4 *NRAS*, 3 *PTPN11* mutations, and 1 each of *FLT3, JAK3, TERT, CBL,* and *CDK2NA*. Ten patients carried only a single mutation, whereas two patients harbored multiple mutations: one with *KRAS* and *CDKN2A* and one with *PTPN11*, *JAK3*, *TERT*, *KRAS*, and *CBL*. The latter patient developed mutations throughout his disease course. No patients carried *NF1* mutations. Nine of the eighteen patients had known cytogenetic abnormalities, including seven with monosomy 7.

Regarding clinical diagnostic criteria, 16 (89%) patients had documented splenomegaly. Of the patients who underwent bone marrow evaluations at diagnosis, all had less than 20% blasts and all had <20% peripheral blasts, with four demonstrating no peripheral blasts. All patients had an absolute monocyte count (AMC) > 1 × 10^9^/L. Thrombocytopenia was noted in 17/18 (94%) patients. Fetal hemoglobin (Hb F) increased with age in all patients for which Hb F was recorded in 15 patients, with 3 unknown.

**Figure 2:**
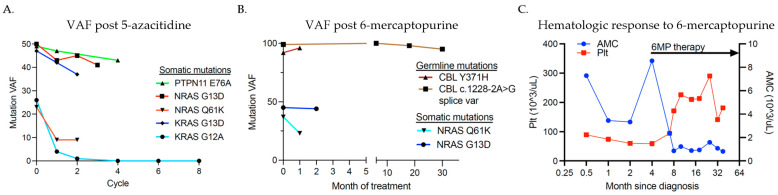
Response to 5-azacitidine and 6-mercaptopurine. Molecular response measured by change in variant allele frequency (VAF) in (**A**) five JMML patients treated with two to eight cycles of 5-azacitidine (0 = pre-cycle 1) and (**B**) four JMML patients treated with oral 6-MP (time 0 = start of therapy). (**C**) Hematologic parameters of a patient with germline *CBL*-mutant JMML before and after the initiation of 6-MP therapy (start of therapy indicated by arrow).

Chemotherapy regimens were known in 16 of the 18 patients with JMML. Six received chemotherapy regimens that did not include 5-azacitidine or 6-MP, three received 6-MP alone, four received 5-azacitidine alone, one received 6-MP followed by 5-azacitidine, five received no therapy, and one proceeded directly to HSCT without bridging therapy. Both patients with germline *CBL* mutations received 6-MP and one then underwent HSCT. Five of the five patients treated with 5-azacitidine and one of four patients treated with 6-MP had a reduction in the mutant variant allele frequency (VAF) (Figure 2A,B).

In general, an increase in bone marrow blast percentage is associated with a statistically significant decrease in the probability of achieving remission (*p* < 0.01) as well as abnormal cytogenetics (*p* < 0.05). Age was also found to have a negative relationship with remission; as age increased, the likelihood of remission (*p <* 0.05) decreased. One somatic *NRAS* mutant patient also continues in active surveillance at 90 months after diagnosis with stable thrombocytopenia, thus not meeting remission criteria. Two patients had germline *CBL* mutations. One patient had substantial improvement in hematologic parameters and splenomegaly with 6-MP alone, although mutant VAF remained unchanged (Figure 2C). The other patient received 6-MP for one month prior to proceeding to HSCT. He had a substantial reduction in his AMC from 14.09 × 10^9^/L to 1.92 × 10^9^/L with 6-MP therapy but no reduction in *CBL* mutant VAF. He achieved clinical and molecular remission after HSCT.

Of the patients with JMML, 8/18 are deceased. Six of the eight deceased patients received HSCT. Three patients died of HSCT complications in the peri-transplant period. One patient died of persistent disease and one of progression of disease to AML. One deceased patient was lost to follow-up and the cause of death is unknown. The two who were not transplanted died of persistent disease and progression to AML. Relapse post-transplant occurred in 3 of the 13 patients transplanted with a median time to relapse of 3.55 months. Transformation to AML was seen in 2 of the 18 patients with JMML (11%) patients, both harboring somatic *KRAS* mutations and one with acquired sub clonal somatic mutations in *PTPN11*, *JAK3*, *TERT*, and *CBL*.

### 3.3. Patient Survival

#### 3.3.1. Survival—Patients Diagnosed with NS-MPD

The median follow-up from the time of diagnosis to the time data were collected was 46.8 months (range of 1.8–90.4 months). All four patients at the time of analysis were still alive. Germline *PTPN11* mutation correlated with age at diagnosis but not survival.

#### 3.3.2. Survival—Patients Diagnosed with JMML

The median follow-up from the time of diagnosis to the time data were collected was 49.4 months (range of 1.3–156.8 months). In patients with a death status, the median follow-up is also survival, and this time averaged 50.0 months (7.7–156.8 months). In patients with a monosomy 7 cytogenetic abnormality, the median time of follow-up or survival at the time of data collection was 47.8 months.

Among all covariates analyzed (sex, germline mutations, somatic mutations, abnormal cytogenetics, splenomegaly, chemotherapy at time of diagnosis, HSCT, age, blast percentage, platelets, and fetal hemoglobin), age and Hb F were found to have associations with the hazard of death, indicating that with each increase of one month in diagnosed age, the likelihood of death increased by 3% (*p* < 0.05) and with each percentile increase in Hb F, there was a 2% increase in the risk of death (*p <* 0.01). The use of cytarabine in chemotherapy given at the time of diagnosis was also associated with a decrease in survival probability (*p* < 0.05); however, due to the limited sample size (*n* = 3), the conclusion has limited statistical power. Age at diagnosis in patients without germline *PTPN11* mutations was not statistically significant to the overall survival. 

## 4. Discussion

In this multi-institutional retrospective study, we reviewed 22 patients with diagnoses of either NS-MPD or JMML. This series provides additional data regarding some of the still unresolved issues of this set of RAS mutation-driven disorders including the disease kinetics of NS-MPD, outcomes of JMML with differing genetic drivers, the impact of cooperating genomic events, and responses to commonly used therapies.

### 4.1. Kinetics of NS-MPD

For all patients with NS-MPD in this series, management consisted of active surveillance as is conventionally accepted [14]. Similar to previous publications [13,25], the time to resolution for these four patients was variable and prolonged. Additionally, hematologic abnormalities appeared in the patients with germline *PTPN11* mutations much earlier than those with somatically mutated *PTPN11,* as has been previously reported [13]. All four patients with NS-MPD were alive at the time of data collection, supporting a conservative approach to such patients.

### 4.2. Outcomes in Genetically Defined JMML Subsets

Patients diagnosed with JMML were organized into five genetically and clinically distinct subtypes of JMML delineated by the driving mutation: somatic *PTPN11*, germline *CBL*, somatic *NRAS*, and somatic *KRAS* [10]. However, patient DNA-sequencing methods were not consistent across the multi-institutional cohort and, thus, patient genetic profiles may be underestimated.

All patients with JMML driven by somatic *PTPN11* mutations received HSCT as is recommended for such patients [19,26,27,28] and two of three survived. Both surviving patients had monosomy 7 but no other identified collaborating genetic lesion. The patient who died had multiple high-risk features (severe thrombocytopenia and elevated fetal hemoglobin) and acquired additional somatic mutations that included *JAK3*, *TERT*, *KRAS*, and *CBL*, and eventual transformation to AML.

Two JMML patients in our cohort had germline *CBL* mutations with a loss of heterozygosity of the wild-type allele and no other identified somatic mutations or genetic abnormalities. Historically, a wide variety of clinical outcomes are noted for germline *CBL*-mutated patients, ranging from spontaneous regression to an aggressive course requiring HSCT [14,25], and currently, there is no consensus for their optimal management. In our cohort, both germline *CBL* mutant patients were treated with 6-MP. Interestingly, despite no improvement in VAF, hematologic parameters rapidly improved with 6-MP therapy. One patient with a germline *CBL* (c.1228-2a>g) mutation demonstrated a response to prolonged 6-MP therapy and has not required further intervention to date (Figure 2C). In a recent case series of 28 patients with germline *CBL*-mutant JMML, only 3 had spontaneous remission, 12 underwent HSCT, and 6 died [16]. In this series, two patients had a germline c.1228-2A>G *CBL* mutation, and both underwent HSCT [16], unlike our patient who continues in remission on 6-MP alone. The second patient in our series had a germline Y371H *CBL* mutation, and given persistent massive splenomegaly, thrombocytopenia, and rising AMC, underwent HSCT. While awaiting, HSCT he was treated with 6-MP monotherapy for one month, which resulted in significantly reduced splenomegaly and a rapid decline in his AMC suggesting clinical benefit. In Hecht et al., six out of eight patients with the germline Y371H *CBL* mutation underwent HSCT, three of whom subsequently relapsed and died of their JMML, suggesting an association with a more aggressive disease phenotype, though additional data are needed [16]. While limited, the responses noted in our two patients suggest that 6-MP may be a reasonable option for patients with JMML and germline *CBL* mutations either as monotherapy during conservative observation or as bridging therapy to HSCT.

Somatic *NRAS* or *KRAS* mutations were found in nine of our patients. *NRAS* or *KRAS* point mutations have been described in 25% of patients with JMML [29], and apart from a small portion of *NRAS*-mutated patients, somatic RAS mutations are usually associated with an aggressive disease course with a high rate of relapse after HSCT [5]. As such, HSCT is generally recommended for children with somatic *KRAS* mutations and most with somatic *NRAS* mutations [9,18]. Our *NRAS*- and *KRAS*-mutated patients all received HSCTs apart from two patients. One *NRAS*-mutated patient who presented without severe disease features continues to have only mild monocytosis and thrombocytopenia after over 13 years of observation. The other un-transplanted patient had a somatic *KRAS* mutation and monosomy 7 and received eight cycles of 5-azacitidine therapy until he achieved remission and currently remains under surveillance (previously reported in PMID 31250550). JMML with a somatic *KRAS* mutation and monosomy 7 has been found to respond well to 5-azacitidine in other reports as well, suggesting this may be a population of JMML patients who can be cured with 5-azacitidine alone, though additional study is needed [22,30,31,32].

### 4.3. Impact of Cooperating Genomic Events

Monosomy 7 is typically associated with poor survival in myelodysplastic syndrome and pediatric AML [33,34,35]; however, its impact on JMML outcomes is unresolved. It has been reported that children with JMML and monosomy 7 have lower presenting leucocyte counts and near-normal fetal hemoglobin, possibly supporting a lower risk profile [5], though the literature is mixed on the prognostic effect of monosomy 7 [19,36]. While three of the seven patients harboring monosomy 7 in our cohort died, monosomy 7 was not associated with decreased survival. This may be confounded by the single patient mentioned with an additional *KRAS* mutation who was diagnosed early in our cohort’s timeframe and continues to do well now, several years post-remission. Nonetheless, a significant association between abnormal cytogenetics and survival should be noted as the majority of our patients with cytogenetic abnormalities, inclusive of monosomy 7, have died regardless of whether they received HSCT. Notably, cytogenetic alteration was also associated with a higher blast count at presentation, possibly reflecting aggressive disease.

### 4.4. Response to Therapy

There have been varied chemotherapy regimens utilized for the initial treatment of JMML. While existing data support the utility of pre-transplant chemotherapy [37], further work is needed to clarify the optimal therapeutic approach, which may vary based on clinical, genetic, and epigenetic factors.

The most commonly used chemotherapy regimens are myeloid-based and may include 6-MP, 5-azacitidine, low-dose cytarabine, or more intense AML-style therapy. Wajid et al. examined the combination of low-dose cytarabine and 6-MP in 33 patients with JMML. There was no difference in overall survival between patients who did or did not receive chemotherapy [38]. As noted above, in our series, two patients with JMML and germline *CBL* mutations had impressive clinical and hematologic responses to single-agent oral 6-MP and tolerated it well. Therefore, 6-MP for this rare group represents a potential option to consider for those who warrant therapy. Interestingly, these patients had clinical and hematologic improvements with 6-MP without a decline in their *CBL* mutant VAF. While this has been previously corroborated [39], further work is needed to understand the mechanisms of response, which do not appear to be related to disease clearance. In addition to the 5-azacitidine case-series data reported above, the Phase 2 trial, AZA-JMML-011, testing the safety and efficacy of 5-azacitidine for the treatment of JMML was recently completed. In total, 18 JMML patients were enrolled, 16/18 completed at least three cycles of therapy, and after three cycles, 61% achieved clinical partial remission [23]. These data suggest that 5-azacitidine monotherapy is an effective bridging therapy for JMML patients prior to HSCT. Regarding the choice of therapy in our cohort, we found an association between cytarabine and decreased survival (*p* = 0.01); however, this finding may have been confounded by disease state (AML transformation, monosomy 7) and the small sample size (N = 3). Otherwise, the choice of chemotherapy at diagnosis was not significantly associated with survival in our study. Nonetheless, it should be noted that all patients treated with 5-azacitidine had a reduction in their mutant VAF, suggesting that it is a reasonable and generally well-tolerated pre-HSCT treatment option that may avoid a more cytotoxic chemotherapy regimen.

### 4.5. Emerging Targeted Therapies

With continued poor overall survival in JMML, novel treatment strategies are still needed. Given the involvement of the RAS/MAPK pathway in JMML, trametinib (a MEK1/2 inhibitor) has been investigated in the first clinical trial in the United States for pediatric patients with relapsed and refractory JMML [40] with preliminary data demonstrating four out of nine patients with clinical responses. This promising result has set the stage for additional studies now in progress to assess the combination of trametinib with azacitidine with or without HSCT in children with newly diagnosed JMML [41].

## 5. Conclusions

This report provides real-world data on a series of patients with JMML or NS-MPD treated at three institutions. Our work provides additional insight into the expected time to spontaneous resolution of NS-MPD in those with germline *PTPN11* mutations and treatment approaches for patients with germline *CBL* mutations where no standard exists. We also add data to the existing JMML and NS-MPD literature on responses to therapy and post-therapy outcomes across differing genetic subsets.

## Figures and Tables

**Table 1:** Diagnostic Criteria of JMML.

Diagnostic Criteria of JMML—WHO ^a^		Diagnostic Criteria of JMML—ICC ^a^
**Category 1: Clinical and Hematologic Features (all features required)**		**Category 1: Clinical and Hematologic Features (features 1 and 2 are present in most cases, features 3 and 4 required)**
1. Peripheral blood monocyte count ≥1 × 10^9^/L 2. Blast percentage in peripheral blood and bone marrow <20% 3. Clinical evidence of organ infiltration, most commonly splenomegaly 4. Absence of the *BCR::ABL1* fusion 5. Absence of *KMT2A* rearrangement		1. Peripheral blood monocyte count ≥1 × 10^9^/L 2. Splenomegaly 3. Blast percentage in peripheral blood and bone marrow <20% 4. Absence of the *BCR::ABL1* fusion
**Category 2: Genetic Criteria (1 criterion required)**		**Category 2: Genetic Criteria (1 criterion required)**
1. Mutation in a component or regulator of the canonical RAS pathway: a. Clonal somatic mutation of *PTPN11*, *KRAS* or *NRAS* ^b^ b. Clonal somatic or germline *NF1* mutation and LOH or compound heterozygosity of *NF1* c. Clonal somatic or germline *CBL* mutation and LOH of *CBL* ^c^ 2. Noncanonical clonal RAS pathway pathogenic variant ^d^ or fusion causing activation of genes upstream of the RAS pathway such as *ALK*, *PDGFR*, and *ROS1*		1. Somatic mutation in *PTPN11, KRAS, NRAS*, or *RRAS* 2. Germline *NF1* mutation and loss of heterozygosity of *NF1* or clinical diagnosis of neurofibromatosis type 1 3. Germline *CBL* mutation and loss of heterozygosity of *CBL*
**Category 3: Must have 2 or more ^e^**		
1. Circulating myeloid and erythroid precursors 2. Increased fetal hemoglobin for age 3. Thrombocytopenia with hypercellular marrow often with megakaryocytic hypoplasia (dysplastic features may or may not be present). 4. Hypersensitivity of myeloid progenitors to GM-CSF (by clonogenic assays or STAT5 phosphorylation with low/no exogenous GM-CSF).		

^a^ Table based on 2022 World Health Organization Classification [8] and 2022 International Consensus Classification of Myeloid Neoplasms and Acute Leukemias [2]. ^b^ Germline mutation in PTPN11, KRAS, and NRAS (Noonan Syndrome) may produce JMML-like transient myeloproliferative disorder. ^c^ Occasional cases have splice-site mutations. ^d^ Such as RRAS, RRAS2. ^e^ Patients who do not meet any of the genetic criteria must meet the following criteria in addition to category 1 criteria. JMML, Juvenile Myelomonocytic Leukemia; LOH, loss of heterozygosity; GM-CSF, granulocyte–macrophage colony-stimulating factor.

**Table 2:** Patient demographics and characteristics.

Characteristic	No. of Patients with NS-MPD, N = 4	No. of Patients with JMML, N = 18
**Median age at diagnosis, Months (range)**	1.4 (0–2)	15.7 (2–59)
**Sex**		
Female	2	3
Male	2	15
**Race/Ethnicity**		
Asian	0	2
Black	1	2
White (Non-Hispanic)	2	4
Hispanic	1	10
**Germline Mutations**		
*PTPN11*	4	0
*CBL*	N/A	2
*NF-1*	N/A	0
**Somatic Mutations**		
*PTPN11*	N/A	3
*FLT-3*	N/A	1
*KRAS*	N/A	5
*NRAS*	N/A	4
**Cytogenetics**		
Monosomy 7	0	7
Other (with or without monosomy 7)	0	5
Normal	4	11
Unknown	0	2
**Initial Chemotherapy Regimens**		
6-Mercaptopurine	0	5
5-Azacitidine	0	5
Cytarabine (combined or alone)	0	3
Other Chemotherapy	0	3
Untreated	4	1
Unknown	0	2
**HSCT**	0	13
**Alive**	4	10
**Median Follow-Up/Survival (Range, months)**	46.8 (1.8–90.4)	49.4 (1.3–156.8)

No., Number; N/A, not applicable; HSCT, hematopoietic stem cell transplant.

**Table 3:** Cohort characteristics at disease baseline and survival status.

			Mutations				Blasts									
Patient	Age	Sex	Germline	Somatic	Cytogenetics	Splenomegaly	BM (%)	Peripheral (%)	AMC (10^9^/L)	Platelets (10^3^/UL)	Hb F (%)	Initial Chemotherapy	Spontaneous Remission	HSCT	Status	Follow-up/Survival (mo.)
**Noonan Syndrome-associated Myeloproliferative Disorder (NS-MPD)**																
1	Birth	F	(NS) *PTPN11*, heterozygous, c.213T>A (p.F71L)	N	Normal	Y	N/A	5	7.7	39	95	N	Ob	N	Alive	1.8
2	3 weeks	M	(NS) *PTPN11*, heterozygous, c.218C>T (p.T731)	N	Normal	Y	N/A	4.2	7.2	45	12.4	N	Ob	N	Alive	10.1
3	3 mo	M	(NS) *PTPN11*, heterozygous, c.317A>C (p.D106A)	N	Normal	Y	6	11	15.6	37	48.6	N	Y	N	Alive	84.7
4	2 mo	F	(NS) *PTPN11*, heterozygous, c.1505C>T (S502L)	N	Normal	N	4.5	0	4.5	19	59.4	N	Y	N	Alive	90.4
**JMML with somatically mutated *PTPN11***																
5	5 mo	M	N	*PTPN11*, c.227A>C (p.E76A)	Monosomy7; der(7)D7S522(7q31)	Y	3.5	2	9.7	208	4.4	5-Aza	N	Y	Alive	8.2
6	3 yo	M	U	*PTPN11*, c.215C>T (p.A72V); JAK3 c.1970G>A (p.R657Q); *TERT*, c.1-124C>T; * KRAS, c.35G>C (p.G12A); * *CBL*, c.1096-1G>C promoter mutation	U	Y	15	12	12.3	26	79.5	6-MP, Fludarabine, Cytarabine	N ^#^	Y	Dead	26.7
7	8 mo	M	N	*PTPN11*, c.226G>A (p.E76K)	Monosomy7; del(7q31); Ring Ch7	Y	3	3	4.5	27	7.4	Directly to transplant	N	Y	Alive	137.3
**JMML with germline mutated *CBL***																
8	12 mo	M	*CBL*, c.1111T>C (p.Y371H)	LOH of wild-type CBL	Normal	Y	0	7	2.3	74	2.6	6-MP	N	Y	Alive	32.5
9	17 mo	F	*CBL*, heterozygous, c.1228-2A>G splice variant	LOH of wild-type CBL	Normal	Y	2	0	5.5	89	3	6-MP	N ^	N	Alive	35.2
**JMML with somatically mutated *NRAS***																
10	14 mo	F	N	*NRAS*, c.181C>A (p.Q61K)	Monosomy7; del(6q); +21	Y	3	0.9	1.4	28	23.4	6-MP, 5-Aza	N	Y	Dead	9.9
11	4 mo	F	N	*NRAS*, c.38G>A (p.G13D)	Normal	Y	0.5	1.6	3.6	17	23.1	6-MP	N	Y	Alive	49.2
12	9 mo	M	N	*NRAS,* c.38G>A (p.G13D)	Normal	N	4	6	3.5	107	5.3	5-Aza	N	Y	Alive	58.4
13	9 mo	M	N	*NRAS*, c.34G>A (p.G12S)	Normal	Y	8	1.6	1.2	26	2.7	N	Y	N	Alive	156.8
**JMML with somatically mutated *KRAS***																
14	10 mo	M	N	*KRAS*, c.35G>C (p.G12A); *CDKN2A* (p16) deletion	Monosomy 7	Y	4.5	0	4.1	69	2.6	5-Aza	N	N	Alive	94.6
15	2 yo	M	U	*KRAS*	Normal	Y	3	0	7.4	83	3	Hydroxyurea, Etanercept	N	Y	Dead	7.7
16	6 mo	M	N	*KRAS,* c.38G>A (p.G13D)	Normal	Y	0.5	1.7	8.7	12	5.3	5-Aza	N	Y	Alive	9.4
17	5 mo	M	N	*KRAS*	U	Y	14	0	39	34	1.2	U	N	Y	Alive	102
**JMML with Other or Unknown genetic abnormalities**																
18	2 mo	M	N	U	Monosomy 7	Y	3	8.4	4.6	17	N/A	Cytarabine, Etoposide	N	N	Dead	1.3
19	21 mo	M	U	U	Monosomy 7	Y	12	4.2	5.2	76	30.3	Cytarabine	N	Y	Dead	14.6
20	8 mo	M	N	N	Monosomy 7	U	10	7.5	2.7	22	U	U	N	Y	Dead	69
21	33 mo	M	N	*FLT3*	Ch 5q, Ch 9q	Y	12	4	9.7	36	84	ARAC, Flucytosine, Cis-retinoic acid	N	Y	Dead	40.1
22	59 mo	M	U	U	Trisomy 8	Y	17	16	3.4	27	U	Imatinib, Cis-retinoic acid, Etanercept	N ^#^	N	Dead	47.3

NS = Noonan Syndrome, del = deletion, der = derivative, Ch = chromosome, LOH = loss of heterozygosity, BM = bone marrow, Hb F = fetal hemoglobin, HSCT = hematopoietic stem cell transplant, Ob = Observation surveillance ongoing, Y = yes, N = no, U = unknown, 6-MP = 6-mercaptopurine, 5-Aza = 5-azacitidine, Follow-Up/Survival = follow-up from time of diagnosis to the time data were collected or survival of deceased patients. * mutations developed during disease course. ^ remains on chemotherapy. ^#^ AML transformation.

## Data Availability

The data presented in this study are available upon request from the corresponding author (M.B.G.) and are not publicly available to maintain patient privacy.

## Supplementary Materials

The following supporting information can be downloaded at: https://www.mdpi.com/article/10.3390/cancers16152749/s1, Table S1: Next Generation Sequencing Panels.
