# Supplementary material for: Observation and Management of Juvenile Myelomonocytic Leukemia and Noonan Syndrome-Associated Myeloproliferative Disorder: A Real-World Experience
Source: Cancers (Basel). 2024 Aug 2;16(15):2749. doi: 10.3390/cancers16152749 (PMC11311611; doi:10.3390/cancers16152749)
Supplement: Supplementary file 1 [file cancers-16-02749-s001.zip › cancers-3022857-supplementary.pdf]

Supplemental Table S1

Texas Children's Hospital Next Generation Sequencing Panel

ABL1	ABL2	AKT1	AKT2	ALK	ANK3	APC	ARAF
ARID1A	ASXL1	ASXL2	ATM	ATRX	BAZ1A	BCL11B	BCOR
BCORL1	BIRC6	BRAF	CALR	CBL	CBLB	CCND1	CCND2
CCND3	CCT6B	CDKN1B	CDKN2A	CDKN2B	CEBPA	CHD4	CNOT3
CREBBP	CRFL2	CSF1R	CSF3R	CTCF	CUX1	DDX3X	DDX41
DHX15	DNM2	DNMT3A	ECT2L	EED	EIF6	ELF1	EP300
EPOR	ERG	ETNK1	ETV6	EZH2	FAT1	FAT4	FBXW7
FLT3	FPGS	GATA1	GATA2	GATA3	GNA13	GNAS	H3F3A
HDAC9	HRAS	HUWE1	ID3	IDH1	IDH2	IKZF1	IKZF2
IKZF3	IL7R	JAK1	JAK2	JAK3	KANSL1	KAT6B	KDM5A
KDM6A	KIT	KMT2A	KMT2C	KMT2D	KRAS	LEF1	MAP2K1
MBNL1	MED12	MGA	MLH1	MLLT3	MPL	MSH2	MSH6
MYB	MYC	MYCN	MYD88	NF1	NIPBL	NOTCH1	NOTCH2
NOTCH3	NPM1	NR3C1	NR3C2	NRAS	NSD2	NT5C2	ORAI1
PAX5	PCBP1	PDGFRA	PDGFRB	PHF6	PHIP	PIK3C2A	PIK3CA
PIK3CD	PIK3R1	PMS2	PPM1D	PRDM2	PRPS1	PRPS2	PTEN
PTPN11	PTPRC	RAD21	RB1	RELN	RHOA	RIT1	RPL10
RPL5	RUNX1	SAMD9	SAMD9L	SETBP1	SETD2	SETX	SF3A1
SF3B1	SH2B3	SMARCA4	SMARCB1	SMC1A	SMC3	SOS1	SRSF2
STAG2	STAT3	STAT5B	SUZ12	SYNE1	TERT	TET1	TET2
TNFRSF14	TP53	TSPYL2	U2AF1	U2AF2	UBTF	USP7	USP9X
VPREB1	WT1	ZBTB7A	ZEB2	ZFHX3	ZRSR2		

MD Anderson Cancer Center Next Generation Sequencing Panel

ANKRD26	ASXL1	ASXL2	BCOR	BCORL1	BRAF	BRINP3	CALR
CBL	CBLB	CBLC	CEBPA	CREBBP	CRLF2	CSF3R	CUX1
DDX41	DNMT3A	EED	ELANE	ETNK1	ETV6	EZH2	FBXW7
FLT3	GATA1	GATA2	GFi1	GNAS	HNRNPK	HRAS	IDH1
IDH2	IKZF1	IL2RG	IL7R	JAK1	JAK2	JAK3	KDM6A
KIT	KMT2A	KRAS	MAP2K1	MPL	NF1	NOTCH1	NPM1
NRAS	PAX5	PHF6	PIGA	PML	PRPF40B	PTEN	PTPN11
RAD21	RAR	RUNX1	SETBP1	SF1	SF3A1	SF3B1	SMC1A
SMC3	SRSF2	STAG1	STAG2	STAT3	STAT5A	STAT5B	SUZ12
TERC	TERT	TET2	TP53	U2AF1	U2AF2	WT1	ZRSR2
